# A Plasmonic Chip-Scale Refractive Index Sensor Design Based on Multiple Fano Resonances

**DOI:** 10.3390/s18103181

**Published:** 2018-09-20

**Authors:** Kunhua Wen, Li Chen, Jinyun Zhou, Liang Lei, Yihong Fang

**Affiliations:** School of Physics and Optoelectronic Engineering, Guangdong University of Technology, Guangzhou 510006, China; ggchenli@gdut.edu.cn (L.C.); zhjy@gdut.edu.cn (J.Z.); leiliang@gdut.edu.cn (L.L.); fongyathong@yeah.net (Y.F.)

**Keywords:** Fano resonances, refractive index sensing, sub-wavelength MIM waveguides

## Abstract

In this paper, multiple Fano resonances preferred in the refractive index sensing area are achieved based on sub-wavelength metal-insulator-metal (MIM) waveguides. Two slot cavities, which are placed between or above the MIM waveguides, can support the bright modes or the dark modes, respectively. Owing to the mode interferences, dual Fano resonances with obvious asymmetrical spectral responses are achieved. High sensitivity and high figure of merit are investigated by using the finite-difference time-domain (FDTD) method. In view of the development of chip-scale integrated photonics, two extra slot cavities are successively added to the structure, and consequently, three and four ultra-sharp Fano peaks with considerable performances are obtained, respectively. It is believed that this proposed structure can find important applications in the on-chip optical sensing and optical communication areas.

## 1. Introduction

Fano resonance was first demonstrated in the atomic system caused by the coherent interference between a discrete state and a continuous state [1,2,3]. The asymmetrical spectrum of Fano resonance is quite different from the Lorentz one obtained from Fabry–Pérot (FP) resonance, which is suitable for an optical filter. These ultra-sharp asymmetrical spectra are preferred in the optical sensing area due to high refractive-index sensitivity and high figure of merit (FOM). Interestingly, Fano resonance is also investigated in the plasmonic metal-insulator-metal (MIM) waveguide, which can overcome the optical diffraction limit owing to the characteristics of surface plasmon polaritons (SPPs) [4,5,6,7,8,9,10]. Consequently, MIM waveguide structures have been considered as one of the most promising ways for developing nano-scale integrated photonic circuits, and they are quite preferred in the optical communication and sensing areas. For example, a bright resonant mode and a dark mode, which are analogous to the continuous and the discrete states, respectively, are generated in the side-coupled dual slot-cavity resonators [11]. Due to the interaction between the bright modes and the dark modes, an asymmetrical sharp transmission peak is achieved in this proposed MIM structure. Besides, an asymmetrical Fano-type spectrum is observed by using dual parallel grooves [12], which are placed on the same side of a MIM waveguide. In these proposed MIM structures, single Fano resonances with high sensitivity and high FOM has been investigated for the sensing purpose [13,14,15,16,17,18,19,20,21,22]. However, considering the development of high integrated photonics circuits, more attention is also paid to the mechanism of multiple Fano resonances in single one subwavelength MIM structure. Therefore, composite configurations based on MIM waveguides, such as groove-cavity composite structure and cascaded grooves structure, have been successively proposed and investigated [23,24,25]. Dual Fano transmission peaks with asymmetrical line shapes are achieved in the infrared wavelength range. In this case, one would also look forward to exploring the on-chip MIM structures to obtain more Fano resonant peaks. 

In this paper, multiple Fano resonances are achieved by employing an end-coupled slot cavity resonator. A slot cavity is perpendicularly inserted between the input and output MIM waveguides, while another horizontal one is placed above the waveguide. Owing to the mode interactions, dual Fano resonances with high sensitivity and high FOM are achieved. Besides, additional slot cavities are added on the middle and bottom of the vertical one, triple and quad Fano sharp peaks with asymmetrical line shapes are also obtained. The performances of the structure are investigated through the finite-difference time-domain (FDTD) method, and it is believed that the proposed structure can find applications in the on-chip optical sensing area. 

## 2. Theory and Analysis

**Figure 1:**
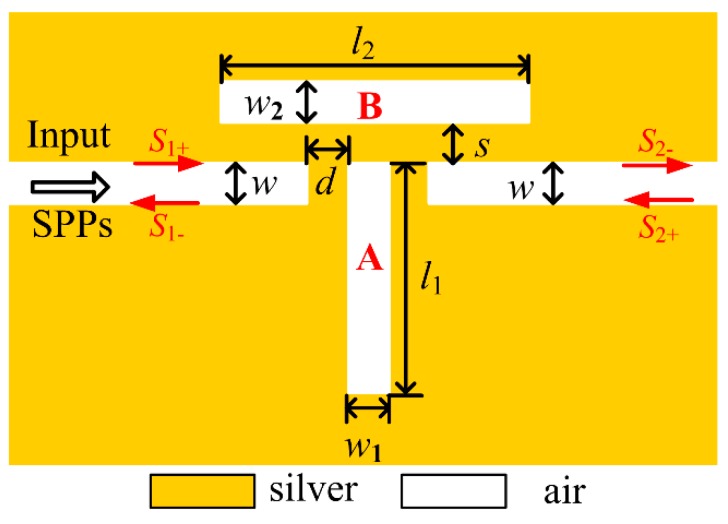
Schematic diagram of the Fano resonant MIM structure, the metal and insulator are silver and air, respectively.

Figure 1 shows the schematic diagram of the plasmonic Fano resonant structure. A vertical slot cavity (named as cavity *A*) is inserted between the input and output MIM waveguides with a coupling distance of *d*. The top of the cavity is aligned with the MIM waveguide. It is well known that the single end-coupled vertical cavity is regarded as a FP resonator that can support the bright modes. Stable standing waves can only build up constructively within the cavity when the following resonant condition is satisfied, based on the principle of a resonant cavity: 4πRe(neff)l1/λ+ϕ=2mπ,m=1,2,3,⋯ [26,27]. Consequently, the resonant wavelength can be derived as:

(1) λ=2Re(neff)l1m−ϕ/2π,m=1, 2, 3, ⋯

where $l_{1}$ is the length of the cavity, *m* stands for the resonant-mode order, and ϕ is the phase shift caused by the reflection at the FP facet. Re(neff) is the real part of the effective index $n_{eff}$, which can be obtained from the dispersion equation [28]: $\varepsilon_{i}k_{m}+\varepsilon_{m}k_{i}\tan h\left(-jk_{i}w/2\right)=0$, where $k_{i,m}=\sqrt{\varepsilon_{i,m}{\left(2\pi /\lambda \right)}^{2}-\beta^{2}}$ is the transverse propagation constant in air and silver, respectively, *w* is the width of the waveguide, and $\varepsilon_{i}$ and $\varepsilon_{m}$ are the dielectric constants of air and silver, respectively. The propagation constant is represented as the effective index of the waveguide: $\beta =2\pi n_{eff}/\lambda$. The optical phase retardation and the propagation loss coefficient of the plasmonic mode are determined by the real part Re(neff) and the imaginary part $Im\left(n_{eff}\right)$, respectively. Since the proposed structure is on a nanometer scale, $Im\left(n_{eff}\right)$ can be ignored and more attention is paid to Re(neff) for obtaining the relative phase. Usually, the bright mode is considered as the resonant pass band, and the dark mode is the forbidden band. Therefore, bright modes can be obtained by the end-coupled cavity *A*. Likewise, a side-couple slot cavity (named as cavity *B*), which is placed over cavity *A* with a coupling distance of *s*, is also an FP resonator, but it will generate the forbidden bands which are regarded as the dark modes. When SPPs are coupled into the slot cavity in the case of a relatively small coupling distance (less than 50 nm), corresponding stable bright and dark modes can build up within cavity *A* and *B*, respectively. Fano resonance occurs and leads to an asymmetrical spectrum with ultra-sharp peaks owing to the mode interference, when the wavelength of the bright mode is close to the dark modes, interference occurs. Accordingly, one must precisely design the lengths for cavity *A* and *B* to access Fano resonance, since the resonant wavelength is proportional to the length. 

Coupled mode theory (CMT) is also employed to analyze the transmission response [29,30]. In Figure 1, $S_{1,\;2\pm }$ stands for the normalized amplitudes of SPPs in the output and input MIM waveguides, respectively, while a and b are the ones inside cavity *A* and cavity *B*, respectively. $Q_{aw}$ and $Q_{ar}$ are the quality factors related to the coupling loss from cavity *A* into the MIM waveguide and the intrinsic loss inside cavity *A*, respectively. $Q_{bw}$ describes the coupling loss from cavity *B* into cavity *A* and the waveguide, and $Q_{br}$ is the intrinsic loss inside cavity *B*. Since SPPs are only launched into the MIM waveguide from left side, the amplitude $S_{2+}$ can be assumed as $S_{2+}=0$, and then the normalized amplitudes a and b of cavity *A* and *B* can be expressed as:

(2) $$\{\begin{array}{c} \frac{da}{dt}=\left(j\omega_{0}-\frac{\omega_{0}}{2Q_{ar}}-\frac{\omega_{0}}{2Q_{aw}}\right)a+\sqrt{\frac{\omega_{0}}{2Q_{aw}}}S_{1+}+j\frac{\omega_{0}}{2Q_{bw}}b\\ \frac{db}{dt}=\left(j\omega_{0}-\frac{\omega_{0}}{2Q_{br}}\right)b+j\frac{\omega_{0}}{2Q_{bw}}a\\ S_{2-}=S_{1+}-\sqrt{\frac{\omega_{0}}{2Q_{aw}}} \end{array}$$

where $\omega_{0}$ is the resonant frequency. Consequently, the transmission T of the output waveguide is derived as:

(3) $$T={\left|\frac{1}{Q_{aw}}\frac{j2\frac{\omega -\omega_{0}}{\omega_{0}}+\frac{1}{Q_{br}}}{{\left(j2\frac{\omega -\omega_{0}}{\omega_{0}}+\frac{1}{2Q_{ar}}+\frac{1}{2Q_{br}}+\frac{1}{2Q_{aw}}\right)}^{2}+{\left(\frac{1}{Q_{bw}}\right)}^{2}-{\left(\frac{1}{2Q_{ar}}-\frac{1}{2Q_{br}}+\frac{1}{2Q_{aw}}\right)}^{2}}\right|}^{2}.$$

**Figure 2:**
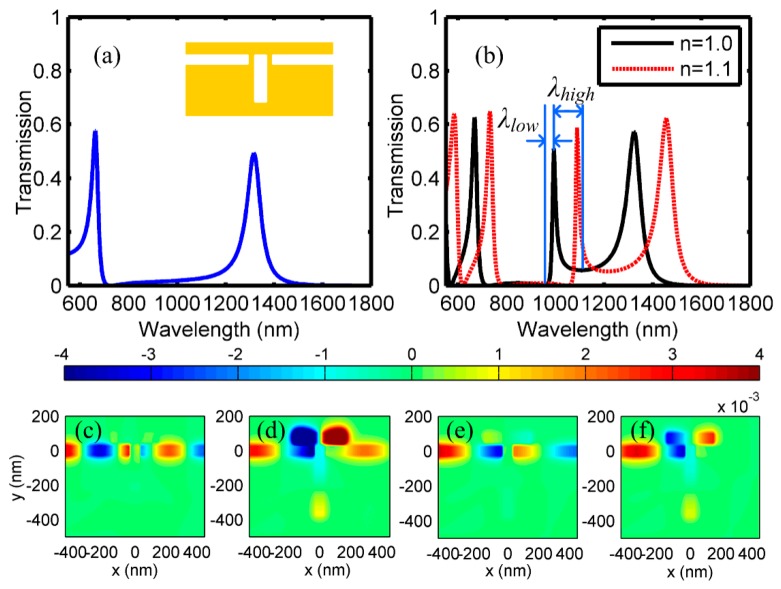
(**a**) Transmission spectrum of the proposed structure with only cavity A, (**b**) Fano spectra of the proposed structure with both cavities (black solid line for *n* = 1.0, red dash line for *n* = 1.1), and (**c**–**f**) The magnetic field distributions at the peak- or dip-wavelengths at 667 nm, 993 nm, 1322 nm, and 958 nm, respectively (corresponding to the spectra in (**b**) with *n* = 1.0).

2D FDTD simulation is employed to obtain the spectrum, since 2D structures can save a lot of running time and hardware resource consumption, but the results would still agree well with the 3D model. In the following FDTD simulation, perfect matching layer is used as the absorption boundary and the mesh accuracy is 5 nm in both *x* and *y* directions. The widths *w* of the MIM waveguide and both cavities are the same (50 nm), and the lengths $l_{1}$ and $l_{2}$ of cavity *A* and *B* are 400 nm and 300 nm, respectively, and the coupling distances *s* and *d* are 25 nm and 15 nm, respectively. The metal and insulator are firstly assumed as silver and air, respectively. Figure 2a shows the transmission spectrum of the proposed structure with only cavity *A*. Owing to FP resonance, there are two pass bands emerging at 667 nm and 1322 nm, respectively. This kind of structure can perform as a traditional optical filter, since high transmission (>0.58) and symmetrical line shape are achieved. After adding cavity *B* above the waveguide, the dark mode and the bright mode will interact with each other, resulting in Fano resonance. The transmission spectrum is shown in Figure 2b with black solid line, which indicates that the previous channel remains at the same wavelength of 1322 nm. In addition, an asymmetrical ultra-sharp transmission peak with a transmittance of 0.51 arises at 993 nm. The transmission at the right side of the peak changes slowly, while the one at the left side has a sharp decline and the dip occurs at 958 nm. The degree of spectral asymmetry *F* is defined as the ration of high/low wavelength transmission bandwidths (peak-to-node): $F=\lambda_{high}/\lambda_{low}$ [31,32,33], as indicated in Figure 2b. For Fano resonance, high degree of asymmetry is preferred and it is calculated as 4.7 based on the results in Figure 2b. Actually, the asymmetry will be affected by the wavelengths of the dark mode and bright mode, and therefore, the lengths of cavity *A* and *B* are the important factors to manipulate the spectral response. Besides, relatively weak Fano resonance is also observed at the wavelengths of 667 nm and 725 nm (corresponding to the Fano peak and dip respectively), since the spectral shape is also asymmetrical.

Consequently, Fano resonance with asymmetrical spectrum is achieved in this proposed structure. To evaluate the performance of the structure that acts as an optical on-chip sensor, the sensitivity, which is one of the most important factors, can be expressed as:

(4) $$S=\frac{d\lambda_{res}}{dn\left(\lambda_{res}\right)}$$

where $\lambda_{res}$ is the resonant wavelength, and $n\left(\lambda_{res}\right)$ is the refractive index of the insulator in the MIM waveguide. When the air is replaced by an insulator with a refractive index of 1.1, the transmission spectrum has an obvious red shift and the transmittances for all the peaks are almost unchanged, as shown in Figure 2b with a red dashed line. In this case, the two Fano resonant peaks appear at 731 nm and 1088 nm, thus, the sensitivities of refractive index are *S* = 640 nm/RIU and 950 nm/RIU, respectively. The detailed magnetic field distributions that correspond to the peaks and dip in Figure 2a are shown in Figure 2c–f. Figure 2c–e represent the SPPs’ propagation details of the resonant peaks at 667 nm, 993 nm and 1322 nm, respectively. Most of the SPPs can propagate through the MIM waveguide, and strong energy distributions can be observed in output waveguide. The magnetic field distribution for the dip at 958 nm is shown in Figure 2f. Interestingly, observed from Figure 2d,f, strong magnetic intensities occuring at both cavities lead to strong mode interference, and then, Fano resonance is achieved.

**Figure 3:**
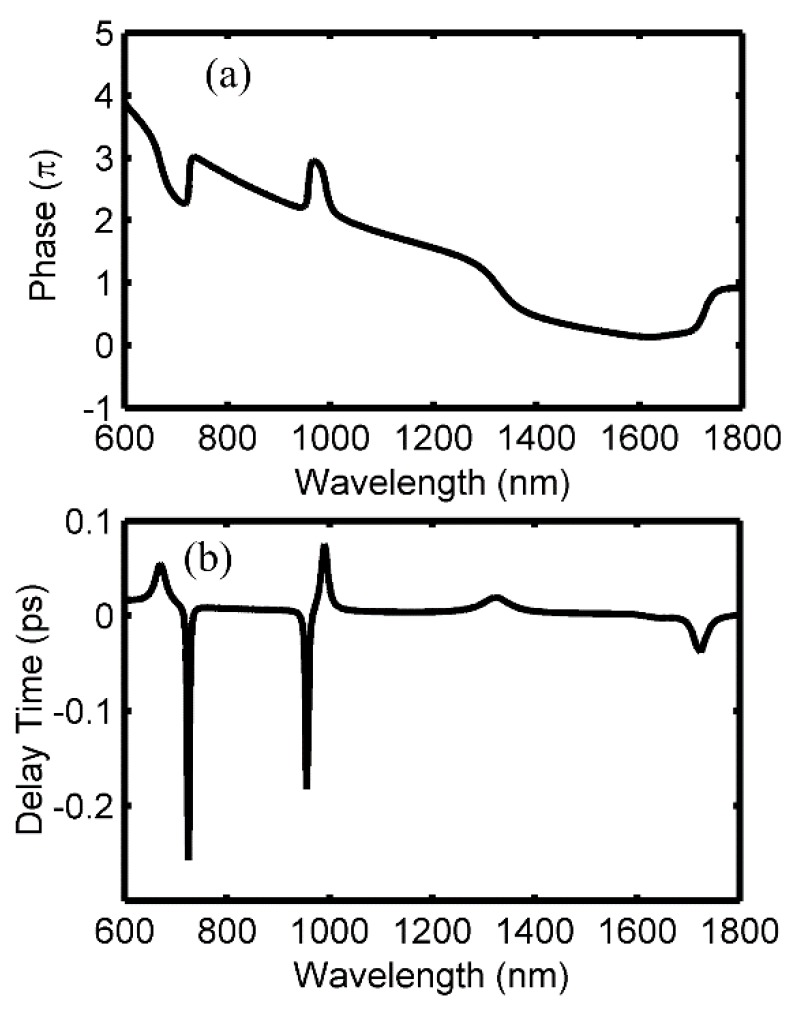
(**a**) Phase response with respect to the wavelength, (**b**) Group delays calculated from phase response.

In addition to the transmission spectrum for the peaks and dips, which will significantly affect the performance of the proposed structure that acts as a sensor, the phase response is also another important factor to explore the applications of the structure. Therefore, to further investigate the Fano resonance, the phase responses and the group delays are also studied, as shown in Figure 3. Accordingly, obvious phase shifts are achieved around the Fano peaks at 667 nm and 993 nm, respectively, and opposite variations are observed at the dips in Figure 3a. Moreover, the phase curve, which changes smoothly at the FP resonant peak of 1322 nm, is quite different from the one of Fano resonance. More details can be excavated from the group delays τ(λ) in Figure 3b, and it can be obtained from the phase responses: $\tau \left(\lambda \right)=-\lambda^{2}d\theta /2\pi cd\lambda$, where θ is the phase shift, and c is the light speed. Obviously, there are large negative group delays within the windows of the Fano dips, and the maximum values of −0.25 ps and −0.18 ps are achieved at 725 nm and 958 nm, respectively. On the contrary, positive delays of 0.05 ps and 0.07 ps are available at the Fano-peak wavelengths of 667 nm and 993 nm, respectively. Consequently, in addition to the sensing applications, one can also develop the fast light or slow light technologies by using the windows of Fano dips or Fano peaks.

**Figure 4:**
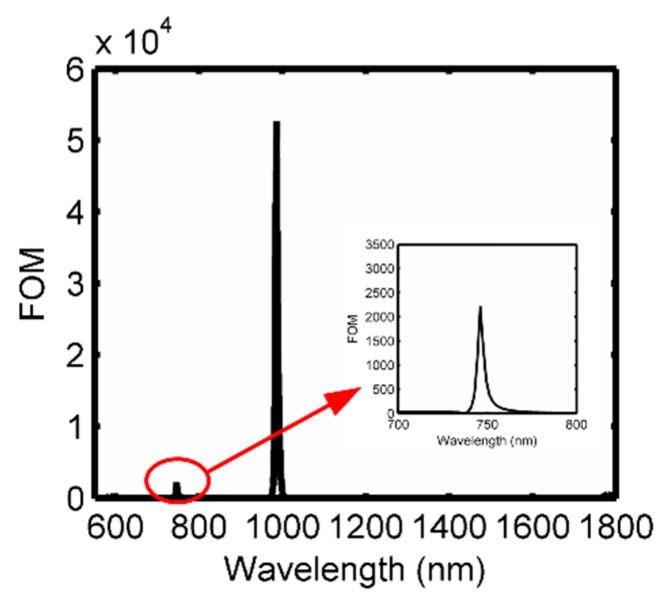
FOM values of the proposed structure, and the sub-graph inside is the magnified area around the Fano dip wavelength.

Furthermore, FOM is also a quality factors to evaluate the performance of Fano resonance. A high FOM value will be preferred in Fano resonance for the sensing applications, and it can be expressed as [11,34]:

(5) $$\mathrm{FOM}=\max\left(\left|\frac{dT\left(\lambda \right)/dn\left(\lambda \right)}{T\left(\lambda \right)}\right|\right)$$

where T(λ) is the transmission, and dT(λ)/dn(λ) is the transmission change caused by the refractive index. Therefore, it can be concluded that an ultra-sharp peak and an ultra-low dip induced by the index changes are preferred for obtaining a high FOM. Based on the transmission spectrum in Figure 2b, the maximum FOM of ~$5.26\times 10^{4}$ is achieved at the Fano peak of 993 nm, and the second largest value of 2.22 × 10^3^ is obtained at another Fano dip, as shown in Figure 4.

**Figure 5:**
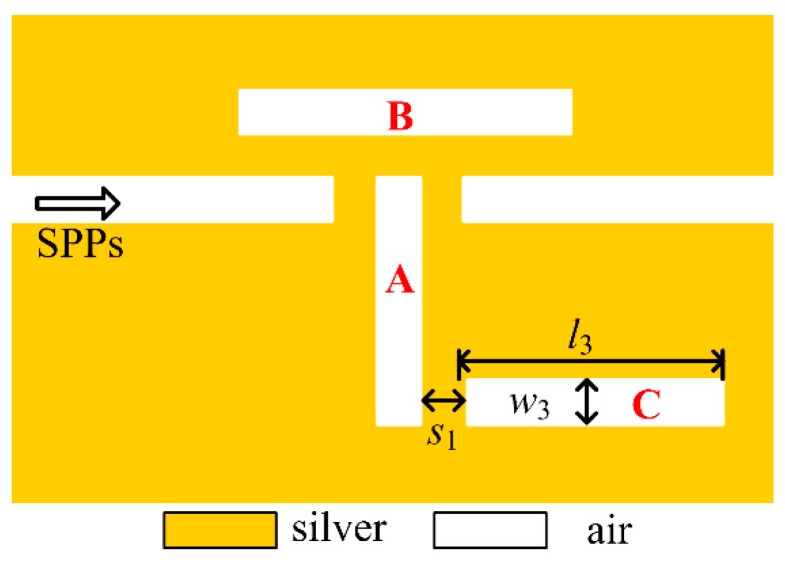
Schematic diagram of Fano resonant structure after adding cavity *C*.

**Figure 6:**
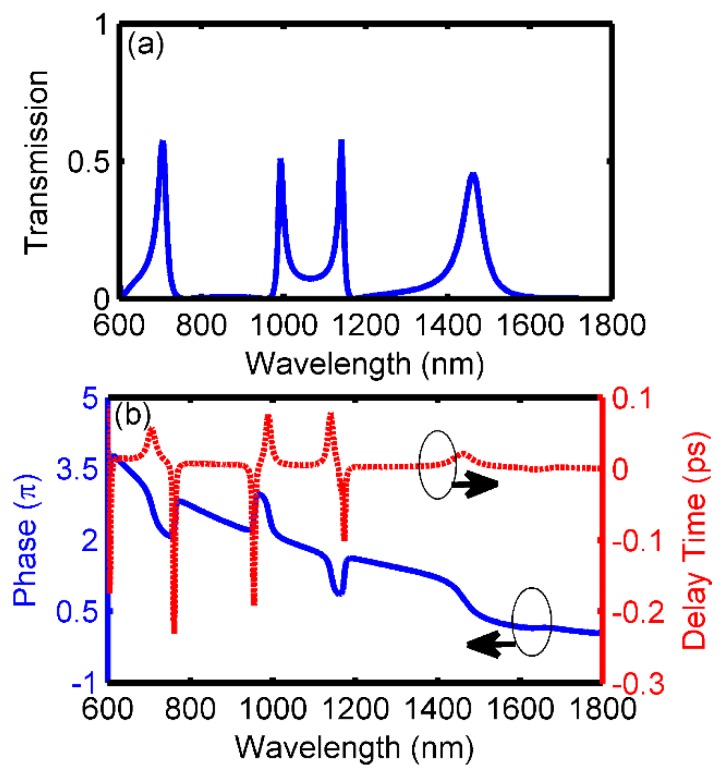
Fano resonances after adding cavity *C* (**a**) transmission spectrum, (**b**) phase responses (blue solid line) and group delays (red dash line).

To obtain more Fano resonant modes in single one structure, cavity *C* is added to the right bottom of cavity *A* with a coupling distance of $s_{1}=10\;\mathrm{nm}$, as shown in Figure 5. Likewise, when the resonant length of cavity *C* is designed appropriately, the resonant mode supported by cavity *C* will interact with the one in cavity *A*, leading to a new asymmetrical Fano peak. After setting the length and width of cavity C as $l_{3}=330\;\mathrm{nm}$ and $w_{3}=40\;\mathrm{nm}$, respectively, the transmission spectrum is shown in Figure 6a. Comparing to the results in Figure 2b, a new sharp Fano peak with a transmittance of 0.58 arises at the wavelength of 1141 nm, while the previous two ones are well remained. For this Fano resonant mode, the asymmetrical dip occurs at the right side of the peak. Then, the sensitivity and the FOM for this Fano peak are calculated as 1090 nm/RIU and $7.63\times 10^{4}$, respectively. More physical phenomenon can be found in the phase responses and the group delays Figure 6b. Obviously, phase shifts are achieved around the three Fano resonant windows, and negative group delays or positive group delays, which are also considered as the distinguishing features for Fano resonance, are obtained for the peaks or dips, respectively. Therefore, triple Fano resonances are investigated in this proposed structure. 

**Figure 7:**
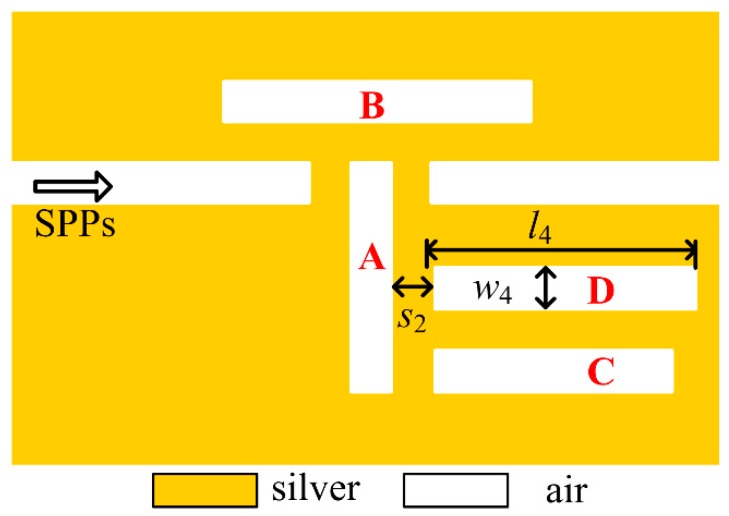
Schematic diagram of Fano resonant structure after adding cavity *D*.

**Figure 8:**
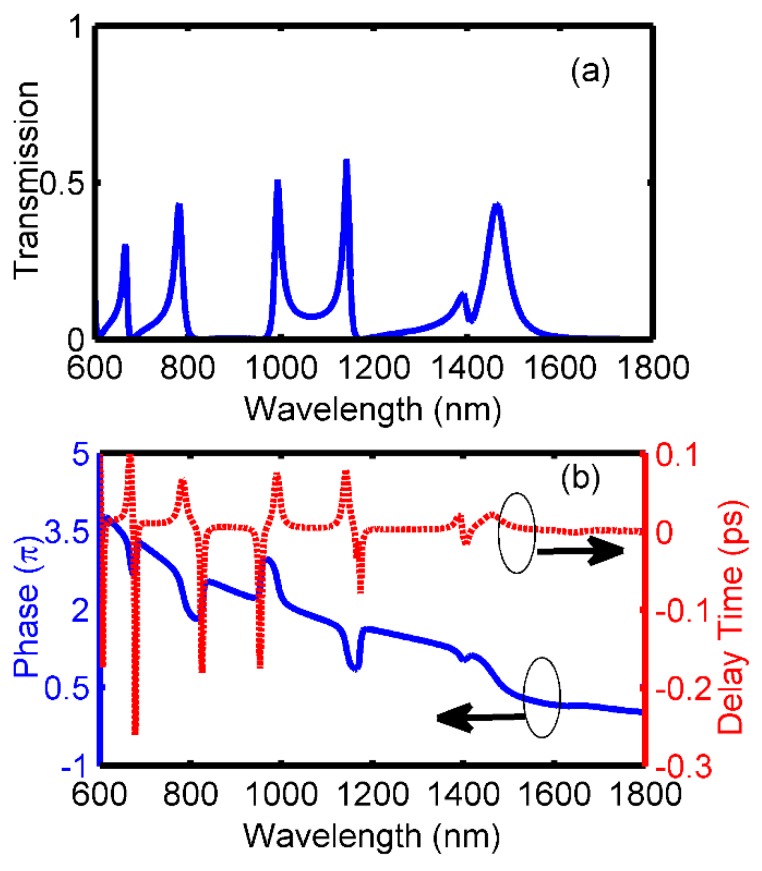
Fano resonances after adding cavity *D* (**a**) Transmission spectrum, (**b**) Phase responses (blue solid line) and group delays (red dash line).

According to the analysis above, if one would like to obtain more Fano peaks and enhance the integration level of the device, it is suggested to design more cavities that can support the proper modes to interact with the ones in cavity *A.* Consequently, cavity *D* is added to the middle position of cavity *A* with a coupling distance of $s_{2}=10\;\mathrm{nm}$ in Figure 7. When the length and width are set as $l_{4}=390\;\mathrm{nm}$ and $w_{4}=40\;\mathrm{nm}$, respectively, and other parameters are unchanged, the transmission spectrum is shown in Figure 8. In this case, there are four Fano resonant peaks with asymmetrical spectral shapes locating at 667 nm, 782 nm, 993 nm, 1141 nm, respectively. Comparing to the spectrum in Figure 6a, one more Fano peak is obtained at 782 nm in Figure 8a. Similar phase responses and group delays are achieved in Figure 8b, which illustrates that phase shifts will be induced by Fano resonances and negative or positive group delays are available at all the Fano dips or peaks, respectively. Specifically, the maximum negative group delays for four dips are −0.26 ps, −0.18 ps, −0.18ps and −0.08 ps, while the maximum positive ones for four peaks are 0.10 ps, 0.07 ps, 0.08 ps, and 0.08ps, respectively. It is believed that this is also another important feature for Fano resonance. 

Compared to the recent work [25], which is a side-coupled tangent-ring Fano resonator, the proposed structure achieves a more compact configuration by using FP resonators. To achieve four Fano resonant peaks, there are five tangent-ring resonators [25], but only three slot cavities are required for the employed structure. Besides, full width half maximum (FWHM) for each peak in this structure is much smaller than that in the previous structure. For example, the FWHM for the Fano peak at 993 nm is 17.5 nm in Figure 2b, but the one for the peak at 920 nm is larger than 200 nm in [25]. Consequently, a higher *Q* factor, which is expressed as λ/FWHM, is achieved in the proposed structure.

## 3. Conclusions

In summary, dual, triple, and quad Fano resonances with asymmetrical spectral responses have been investigated in this proposed structure owing to the mode interactions. Firstly, dual sharp Fano peaks by using cavities *A* and *B* are available at the wavelengths of 667 nm and 993 nm, respectively. High sensitivity of 950 nm/RIU and high ~$5.26\times 10^{4}$ are investigated for the Fano peak. Then, three and four Fano resonant peaks emerge in the wavelength range of 600–1800 nm after adding cavities *C* and *D*, respectively, and considerable performance is also achieved. Moreover, negative and positive group delays have also been investigated in the resonant windows. Compared to the side coupled grooves, more Fano resonant peaks are achieved in the proposed slot-cavity structure by properly adding more slot cavities. Single or dual Fano peaks are achieved in the side coupled grooves, however, four peaks with considerable performances are obtained in the proposed structure. The integration and the performance of the structure are improved. Therefore, the proposed device can be used in the on-chip optical sensing, slow light and fast light areas, and it is believed that the structure will be beneficial to the development of nano-scale integrated photonics.
